# Introduction: contextualising “rights” in sexual and reproductive health

**DOI:** 10.1186/1472-698X-11-S3-S1

**Published:** 2011-12-16

**Authors:** Hilary Standing, Kate Hawkins, Elizabeth Mills, Sally Theobald, Chi-Chi Undie

**Affiliations:** 1 Institute of Development Studies, University of Sussex, BN1 9RE, UK; 2 Liverpool School of Tropical Medicine, Pembroke Place, Liverpool, L3 5QA, UK; 3 Population Council, General Accident House, Ralph Bunche Road, P.O. Box 17643, 00500 Nairobi, Kenya

## 

The idea for this supplement arose from discussions among a set of research partners associated with the Realising Rights Research Programme Consortium (RR RPC), an international partnership funded by the UK Department for International Development from 2005-10 that focused on neglected areas of sexual and reproductive health and rights (SRHR) [1]. In the Consortium, work on rights has been concerned with ways of bridging the gap between international legal human rights frameworks as applied to SRHR, and how these play out for actual people ‘on the ground’. We noted that there was a well-developed *international* language of human rights in relation to sexual and reproductive health, accompanied by significant international advocacy efforts stretching back several decades [2-4]. However, SRHR remained controversial and contested; sexual rights in particular are poorly understood by many policy actors, they are not easy to operationalise ‘downstream’ in policies and programmes, and their place and relevance in people’s day to day lives have been much less explored [5-7]. The papers in this volume are one contribution to the task of laying out why it is important to fill this gap and what the analytical challenges are in doing so.

We decided, therefore, to focus our thematic work on rights on the challenges of contextualising and operationalising the concept in different local and national domains. The aim was to start from the perspective of lived experience, rather than from an abstract, universal concept of rights. This perspective reflects our thinking and discussions about rights in terms of grounded and localised understandings. These suggest the need to go beyond the concept of the abstract legal individual around which much human rights thinking is framed [3,8,9].

For the most part, SRHR literature falls into three broad categories: conceptual and analytical exploration of what rights to health are and how they are constructed [10-13]; rights-based approaches in policy and programmes [14-16]; and advocacy focused pieces for specific areas of rights, e.g. rights of People Living with HIV and AIDS [17,18]. These approaches have greatly enhanced our understanding of the relationship between SRH and rights, yet we still know remarkably little about the relevance of “rights” in people’s everyday lives. There is currently not very much literature which addresses this question in different contexts. This collection seeks to begin filling the gap by analysing the lived experiences of SRHR from diverse groups of women and men from Bangladesh, India, Kenya, Ghana, Nigeria and South Africa and more globally.

Authors in this collection draw on the rich range of analyses of rights and responsibilities, noted in the previous paragraph. They also draw on the work of a number of African researchers who have drawn attention to disjunctions between the language of legal individualism coming from western jurisprudence and the more collective notions of self and personhood which characterise other societies [19,20]. These include anthropological or ethnographic accounts which draw attention to contextually specific articulations of human entitlements [21].

In many contexts, the language of rights is not necessarily how people frame their understandings of reproductive and sexual wellbeing or their sense of entitlement to it. However, we know much less about alternative framings of reproductive health, sexuality and wellbeing that do not take rights as their starting point and how they intersect with different dimensions of well/illbeing, such as poverty, stigma and discrimination. Yet they are critical from the perspective of interventions through public health, law and social policy. At the same time, the international discourse of human rights has been profoundly influential in policy making and programming for SRH and has permeated national and local debates and practice in many places. The interplay between these discourses, debates and practices on the ground is an exciting field for exploration by researchers and SRH rights advocates. There is much to learn from this for developing contextually grounded rights-based approaches.

The Cairo Conference of 1994 on Population and Reproductive Health (ICPD) provides the omnipresent background to discussion about SRHR. 179 nations are signatories to the Programme of Action, which shifted the emphasis decisively from a population-based focus on reducing fertility rates to a comprehensive approach to reproductive health. This laid out a broad development agenda, encompassing gender equity goals along with a much wider scope of sexual and reproductive health services [22,23].

The ICPD Programme of Action is framed within a human rights-based understanding of reproductive health rights which was developed over previous decades by women’s health activists and advocates, both national and international [2]. ICPD has set much of the tenor of the debate about SRHR since then, despite the fact that implementation has been very uneven and there have been major challenges from conservative political forces, from the fragmentation of global health architecture into single disease-focused initiatives, and from the complexities and practical difficulties of SRHR programming [2]. The papers in this supplement owe much to the epistemic shift that ICPD represents.

However, there is also a need to look towards upcoming targets and beyond. The target date for meeting the Millennium Development Goals is coming soon (2015) and critics argue that progress has been limited as these goals have not sufficiently taken reproductive rights into account, nor provided a sufficiently enabling environment to promote and support these rights [24]. Legal frameworks and goals do not provide the full picture of SRHR from the perspectives of people’s lived experiences. As we have seen with the international experience of gender mainstreaming, commitments and treaties at international and national level can mutate and even evaporate as they are interpreted and implemented (with varying degrees of resources) at local level [25]. There is a need to more fully understand the framing of rights by people struggling to achieve social justice and a better standard of life and bring this learning to the realities of implementation of international policies in different national contexts. As the papers illustrate, this can be a challenging process in the complex and contested arena of SRHR.

The first paper, by Chi-Chi Undie and Chimaraoke Izugbara [26], throws down a challenge to concepts of SRH rights as properties of abstract individuals. They argue that the hostility to rights concepts encountered in many African contexts has its roots in strong community-based understandings of entitlements and responsibilities that are in tension with the perceived individualism of human rights discourses. They draw upon anthropological research among the Ubang and Igbo groups in south-eastern Nigeria as well as on contemporary debates about sexual and reproductive health in the Africa region to unpack these tensions and consider ways of reconciling them [26].

They focus on how rights are constituted indigenously through concepts of belonging and how important local constructions of sexuality, gender and the body are in informing and creating entitlements in local cultures in Nigeria. Their aim is to provoke debate and stimulate reflection on these diverse ways of constructing personhood and entitlement. They find that rights in the two indigenous contexts in question are always socially embedded – they are activated through social relations, not through the fact of existence itself. The authors show that the two cultures have indigenous concepts of rights which are grounded both in a sense of human-ness (which is shared with modern human rights discourse) and a belonging-ness to particular communities of human beings through gender, generation, lineage membership etc. They describe the social, economic, political and personal entitlements which flow from this belonging-ness. They note the ways in which these have deep implications for sexual and reproductive entitlements within the society; the much stronger emphasis on social rather than biological parenthood, for instance, creating different dynamics around reproductive choices [26].

Undie and Izugbara suggest that the often antagonistic reaction that activists for citizens’ rights meet in Africa has less to do with the concept of rights than with how activists frame the issue of rights. These framings usually diverge from local understandings of rights, entitlements and responsibilities. A completely individual approach to rights, they argue, risks disregarding existing socially located rights and privileges in many African societies. This leads to a lack of engagement with social locations, limiting the impact of rights movements in Africa. They note that this critique also has resonance with recent international commentaries on sexual and reproductive health rights as human rights [26]. For example, Corrêa, Petchesky and Parker [27] reject dichotomous understandings of the individual and the community. They argue that rights are not either completely communal, or completely individual. Rather, they encompass singularity and interdependence, being both individual and social as are human beings. In the context of SRHR therefore, individuals must be guaranteed social rights, such as to health care and livelihood, to function as individual human bodies.

What does this critique imply for SRHR advocacy? Undie and Izugbara argue that there is potential for building on local concepts of rights and associated duties to safeguard entitlements and these can be openings for stimulating dialogue about other kinds of rights and the social locations in which they belong [26]. This suggestion – that communally located rights can be a pathway to transforming debate and leveraging influence in terms of personal bodily rights – is interesting and provocative. It raises many questions, both about strategies for doing this and the potential for reconciling what may be very different conceptions of rights, for instance in the context of more controversial areas such as safe abortion.

In her paper on adolescent women’s sexual and reproductive health in urban slums of Bangladesh, Sabina Faiz Rashid addresses the huge gap between international legal human rights frameworks as applied to sexual and reproductive health rights and the realities for these young women [28]. Urban slum populations are increasing rapidly around the world. Their populations are often disproportionately young, and slums are locations simultaneously of new livelihood opportunities and new insecurities. Drawing on her extensive ethnographic fieldwork in slum areas of Dhaka city, Rashid provides a detailed, nuanced examination of the reproductive lives of a representative sample of young women living in the slums. The realities of their lives include lack of or very limited access to basic facilities such as clean water and sanitation and health care, flimsy shelter and frequent risks from fires and flooding, and extreme personal vulnerability due to crime and high levels of violence, both structural and interpersonal. Age, gender and poverty exacerbate individual insecurity in slums. Rashid’s research shows how the lived experiences and decisions made about marriage, relationships, bearing children and terminating pregnancies are far removed from the formal SRH rights endorsed at national level, where the Bangladesh government is a signatory to ICPD and various international human rights frameworks [28].

Rashid’s study points to the huge importance of personal insecurity in determining the trajectories of these young women’s sexual and reproductive lives. In the absence of competent policy implementation and the rule of law, slum areas are governed by gang leaders and other local strongmen and landlords. There are links with organised crime and often corrupt local politicians. Young women experience routine sexual harassment and may be caught up in gang feuds through their male family members where women can be victims of gang rape. One outcome of this is a resort to very early marriage, as young as 14 years of age, as a way of protecting adolescent women and providing them with some personal security. However, this security is often short-lived as marriages are unstable – husbands may desert them and move elsewhere or initiate other relationships. Despite significant investment in SRH in Bangladesh and a large family planning programme and some legal access to early termination of pregnancy, women in slums continue to have inadequate access to these needed services, with the State neglecting the situation of slum dwellers. At the same time, bearing a child, particularly a son, continues to be a critical route to social and familial acceptance, whatever the personal reproductive wishes of the young women themselves [28].

In a setting defined by patriarchal structures, strong cultural pressures on female behaviour, and lack of economic power, Rashid shows how women make rational decisions about their sexual, marital and childbearing options. They may be aware of other options but act realistically in the context of the constraints on their lives. One interesting area where they do sometimes choose to exercise a different sense of their ‘rights’ is in the increasing trend towards ‘love marriage’. The slum both curtails their personal autonomy and provides new opportunities, such as the capacity to have boyfriends and meet potential husbands. An emerging language of ‘right’ to personal choice in marriage is developing. However, Rashid’s evidence suggests that these marriages are not any more stable than those arranged by families [28].

This paper demonstrates graphically how the individual politics of reproductive and sexual behaviour and associated ‘rights’ are embedded in larger socio-cultural, political and economic inequalities. Rashid argues that reproductive rights and well-being cannot be meaningfully secured without first addressing the context of extreme poverty and violence threatening adolescent women’s basic human rights and survival. Without this, there will continue to be a disconnect between the discourse of international SRH human rights and the realities within which large numbers of women live their lives and make their day-to-day decisions [28].

In her paper examining a decade of grass-roots experience of working for maternal health rights in the state of Uttar Pradesh, northern India, Jashodhara Dasgupta highlights the importance of going beyond normative concepts of rights and frameworks of rights-claiming [29]. She argues for the importance of actor-focused perspectives on rights in any social struggle to negotiate claims based in social justice and human rights approaches. Dasgupta’s organisation, Sahayog, works with very poor and marginalised rural women in a part of India with one of the highest levels of maternal mortality in the world. Using theoretical frameworks on public accountability and on gendered rights claiming, she draws out lessons for rights claiming strategies in environments characterised by high levels of social, economic and political inequalities [29].

Again, along with our other authors, she notes the importance of understanding how the structures of choices within which people perceive, evaluate and act are constituted within social relations rather than being the properties of autonomous individuals. The context is one where poor women from marginalised communities such as Dalits (the former ‘untouchables’) experience powerlessness in relation to their formal entitlements to health care supposedly provided by the state as a universal benefit. They are often treated disrespectfully, humiliated or denied their entitlements to maternal health services – the duty bearers fail in their responsibilities and the structures of accountability are weak or held in disregard by those who should be held to account [29].

This paper draws upon the author’s deep engagement through Sahayog, the organisation she co-founded 1992, with the problematic of rights-claiming in such a setting. Using first-hand accounts and documentation of successive campaigns, of debates among the various allied civil society groups involved, and strategies developed at different points in time, Dasgupta asks why the issue of maternal deaths has never become a ‘political’ issue, why it has been so difficult, despite scattered local gains, to create a climate of greater accountability within the health system and bureaucracy, and what can be done to secure basic rights in maternal health [29].

She describes four phases of strategic thinking and action. The first phase was concerned with unearthing cases of maternal deaths due to negligence and publicising them through campaigns and media accounts. The second phase built on the recognition that this strategy simply produced denial and defensiveness and led to a modified strategy of facilitating a process of dialogue with state officials and deepening the evidence base on avoidable death cases. However, the collective effort had few tangible results in terms of increased accountability. In the third phase, the alliance of civil society organisations took a further step to build a grassroots forum of local women, using a rights and empowerment methodology to build their capacity as active citizens to claim their maternal health rights. This has become a large, articulate body of women, many of whom have become politically active. The fourth phase has been to build a stronger coalition among the key stakeholders, based on a consensus that the high level of preventable maternal mortality in Uttar Pradesh is unacceptable [29].

This is a rich seam of experience from which important lessons can be learned about strategies for engagement, and in particular, how to effect a shift to a social justice based understanding and practice of accountability. As Dasgupta notes, creating a voice for a hitherto voiceless community is in itself a critical shift which has far-reaching effects on the self-esteem of the claimants and on their understanding of their ‘right to articulate rights’ [29]. And it does have some impact on the institutions that have a duty to meet their claims. But there is a long way to go to shift the huge accountability deficit in such grossly unequal settings.

Dina Siddiqi’s paper on sexuality, rights and personhood examines what happens when normative, ‘global’ discourses on rights which assume clearly individuated sexual identities, confront the messiness of ‘local’ realities in Bangladesh [30]. Drawing on recent research conducted in Dhaka city on changing understandings of sexuality among students, workers in the garments industry and self-identified sexual minorities, Siddiqi examines the local realities of identity politics as they manifest in debates over the naming of male, and particularly male-to-male, sexualities and forms of desire. She considers the implications of the emerging tensions for both public health and sexual rights activism [30].

She points out that globalized identity categories such as Men who have Sex with Men – which are part of the discourse of public health, the state and donor agencies, and gay/lesbian – which are part of the discourse of human rights activists and international non-governmental organisations (NGOs), are too narrow and fixed to capture the ways in which gender and sexually nonconforming persons understand themselves in Bangladesh. In the South Asian context, there has always been a developed cultural language of male non-conforming sexualities which does not map onto these globalized categories [30].

Siddiqi’s paper provides a fascinating case study of how the development of funded programmes to support both public health aims and rights-claiming has resulted in a bureaucratisation of sexual identities which is at odds with people’s lived experience. Individuals whose sexuality is fluid or culturally self-defined in different ways, find themselves isolated or unable to obtain services if they do not conform to the ‘correct’ category. At the same time, she notes the way in which social class has opened up an increasing gap between an elite, educated group that has access to the internet and to global social networks that coalesce around identity categories such as ‘gay’, and poorer men who lack this kind of access [30].

She argues that a simple politics of recognition is inadequate to the task of promoting health and human rights for all as it excludes individuals who do not necessarily connect their sexual practices with a specific sexual or social identity and takes no account of the deep structural inequalities that define an individual’s capacity to participate in a global citizenship of identity-based rights [30].

The paper by Cheryl Overs and Kate Hawkins examines the connections and disconnections between different framings of sex workers’ sexual and reproductive health and rights and the ways in which international law is interpreted in policing and regulatory practices [31]. They note the tension between those actors concerned with upholding the rights of sex workers in order to reduce vulnerability to HIV and other forms of ill-health, and those who see sex work as itself a violation of rights. This tension has been played out particularly between international-level frameworks and resolutions linking sex work, human rights and public health, and national laws and policies where criminalisation of sex workers (or their clients in some countries) is widespread [31].

In a detailed review of sources from a wide range of actor perspectives, including material generated by sex workers themselves, the authors assess the strengths and weaknesses of different ways of framing sex workers’ rights, and suggest pathways for a rights-based approach to sex work in the context of sexual and reproductive health and the appropriate roles of national agencies [31].

Sex work is an area fraught with moral contention. As the authors note, discussion about it is often tied up with narratives about disease control, harm reduction; female virtue and empowerment, migration and slavery. The authors describe findings of high levels of abuse of basic rights of individuals involved in sex work, often in the name of ‘rescue’ or of concerns with ‘sex trafficking’ of vulnerable women and girls. Stigma and discrimination are rife. Sex workers and their advocates argue that sex work is a legitimate occupation that should be recognised as such. Decriminalising it would enable sex workers to realise their sexual, reproductive and other rights by removing their vulnerability to exploitation. Using the law against sex work limits their access to services and creates the unsafe working conditions that drive the transmission of sexually transmitted infections and HIV and produce unwanted pregnancies. It also constitutes a denial of human rights to a livelihood and to citizenship [31].

They note from their review of international legal frameworks, that these are often at odds with grounded understandings of entitlements as well as being frequently inconsistent with each other. They argue that sex workers’ rights claims are based on experiential understandings of human rights grounded in social justice rather than on technical knowledge of international human rights conventions and laws and the respective rights and obligations that they confer.

Through their examination of recent documents and manifestos produced by organised groups of sex workers in many countries, Overs and Hawkins show that what links them is a common call for the decriminalisation of sex work and broader concepts of sex worker rights. Decriminalisation could enable commercial sex to be conducted in safe workplaces, reduce violence and increase access to health services. It could provide an opportunity to realise sex workers’ sexual and reproductive rights. Recognising sex work as an occupation should bring it into the purview of labour law and link it to workplace health and safety [31].

But good regulations, effective health programmes and equitable policy do not automatically begin when criminal laws are removed, even in rich and well governed countries, let alone where regulatory systems generally are not well organised. If criminal laws are replaced with discriminatory or inappropriate policy and law, the health and safety conditions in the sex industry could actually be made worse. They conclude that governments need to move to a more evidence-based formulation of policy in this area, collecting better data and examining more clearly the consequences of different regulatory approaches [31].

In their paper on the ways in which rights language has been framed in South Africa within HIV treatment programmes, Hayley MacGregor and Elizabeth Mills draw attention to the complexities of HIV-related rights discourses and practices in the context of high levels of poverty and socio-economic, race and gender inequalities [32]. The adoption of the new Constitution in 1996 ushered in a raft of progressive legislation related to sexual and reproductive health rights and in accord with international frameworks on SRHR. This has had a major impact on the availability of services. However, HIV rates in South Africa are some of the highest in the world, and women have been disproportionately affected by the epidemic, with an estimated 20% of women in the reproductive years now HIV-positive. There are thus considerable challenges in meeting SRH needs and peoples’ legitimate expectations [32].

In 2003, as part of advocacy work by the South African Treatment Action Campaign (TAC) to exert pressure on the government to introduce anti-retroviral therapy treatment programmes, a number of HIV-positive women participated in a Body Mapping initiative to demonstrate how HIV has affected their bodies and their lives. MacGregor and Mills interviewed some of these women five years later to explore their subsequent experiences and what had changed in the intervening period. Their paper examines these experiences and reflects on the sometimes unintended ways in which the emergent discourse of rights and responsibilities has played out in their lives. In particular, it explores what practical significance rights have in their everyday lives and the ways in which the women, who have a strong history of AIDS activism, have construed their rights and responsibilities with regard to sexual relationships and fertility desires and decisions [32].

Through in-depth interviews with the women, they examined two major issues for HIV-positive women: the problematic of disclosure of HIV status to potential partners and reproductive choice. They found that the language of personal responsibility emphasised in the treatment programme, while very much accepted, created significant dilemmas for the women in negotiating relationships. They lacked the power to insist on the use of condoms and feared that disclosure of their status would lead to stigma, abandonment, loss of financial support or possibly violence. They adopted different ways of managing this dilemma, for instance considering the offer of a condom as being the equivalent of disclosure. MacGregor and Mills suggest that pressure to disclose status can inhibit rights that have meaning for women, such as a right to silence [32].

In terms of reproductive choice, women confronted often very negative attitudes from health care providers regarding childbearing. The tendency to focus on contraceptive provision has reinforced wider social attitudes that HIV-positive women should not have children, reducing their capacity to make informed choices in line with their right to decide autonomously. As the authors note, in settings of structural inequality, there are tensions between concepts of rights and notions of individual responsibility. The latter can translate on the ground into moralistic directives that override rights [32].

As in other papers, the authors note the tension between sexual and reproductive health rights enshrined in international law and now codified in national legislation, and the lived reality of these women. They argue that this is in fact a tension between the liberal model of rights that underpins the legislation and that assumes ‘sameness’ between women and between women and men, and the huge structural inequalities that shape the experiences of different women and of women and men. Women learn to ‘make do’ in the face of inequalities and structural constraints. It is these inequalities that, in the context of sexual and reproductive choices, lead to different perspectives, beliefs and desires [32].

At the same time, they also note another tension, which resonates with the arguments of Undie and Izugbara’s paper [26]. The South African legal system is pluralistic – it also recognises customary law with its historically different constructions of the individual and more communal conceptions of entitlements and responsibilities. These complicate further any settled consensus on SRH rights [32].

Finally, Rose Oronje, Joanna Crichton, Sally Theobald, Nana Oye Lithur and Latifat Ibisomi examine the challenges of operationalising SRH rights in sub-Saharan Africa, and the strategies that have been employed to do this in different political and legal settings [33]. Their concern is with how different stakeholders mobilise and negotiate to get issues that are often highly contested onto policy agendas. This is an under-researched area. At international and regional level, they focus on progress with the African Union’s Maputo Plan of Action (MPoA) on SRHR, launched as a continent-wide framework for policy on SRH in 2006. At national level, they look at the experience of getting Ghana’s Domestic Violence Act (2007) and the 2006 Sexual Offences Act in Kenya through the respective national parliaments [33].

These plans and policies have each generated considerable controversy and opposition that is frequently rooted in social and cultural conservatism. Issues such as safe abortion and legislation on sexual behaviour arouse strong feelings and resistance. This paper shows how challenging it has been to make headway against very entrenched sets of values particularly where they impinge on men’s sense of patriarchal entitlements. The case study of the Maputo Plan of Action highlights the very difficult legal and constitutional settings in many African countries in respect of implementing SRHR, despite the fact that these countries are signed up to the MPoA and previous conventions that support SRHR. The national case studies demonstrate how difficult it can be to change existing laws on issues such as sexual violence [33].

Resource shortages for SRH programmes (particularly in the context of often competing programmes focused on HIV and AIDS), lack of political leadership and a frequent hostility to the language of rights were also found to be key constraints to operationalising SRHR. Women’s sexuality was often framed in negative and personalised ways within cultures where gender inequality is deeply entrenched, raising questions about how to engage men and influential stakeholders in countering such framings. Rights language can close doors to influence [33].

In the context of national policy influencing, the authors note the need to use a very wide range of advocacy strategies in creating a more conducive climate for policy making and legislation.These include long-term coalition building with a wide range of stakeholders, public meetings and campaigns, workshops, placing of articles and stories in the media, and judicious use of research evidence and statistics with key stakeholders in parliament and other national bodies. In terms of the legislative process, they draw attention to the need for an in-depth understanding of government legislative processes and a preparedness to make trade-offs or compromises in order to get at least some of the desired agenda through.

The authors discuss four strategies that advocates have used to leverage policy influence. Forging strategic alliances, bringing together the widest range of actors from both outside and within parliaments, is critical to any attempt to introduce or change policies. Strategic ‘reframing’ is a way of presenting issues through concepts that are likely to have more positive resonance, for instance reframing SRHR in terms of its contribution to national development. Searching out and working with key players in government who may be more sympathetic was found to be effective in both the Ghana and Kenya cases. Strategic opportunism is a way of taking advantage of entry points that may emerge unexpectedly, such as where a debate opens up in a cognate area and allows space to include SRHR. These are strategies from which advocates starting out on this path can usefully learn [33].

These papers raise many important and provocative issues for researchers, activists and service organisations working in the field of SRHR. All demonstrate the overriding importance of wider contexts of structural inequality in constraining rights realisation at both individual and community levels. Poverty, class, gender, ethnicity and other markers for disadvantage can pose major challenges to implementation of human rights law, regardless of national policy. Poor and marginalised people construct their sense of the possible within these constraints. They may not necessarily be unaware of or lack an understanding of these rights but they act – ‘make do’ – within the social, economic and political constraints that bear upon their lives. At the same time, they may create their own versions and meanings of rights, using these to negotiate new social contracts within the changing realities that they experience. The papers provide examples of how ‘grievances’ can become reframed as rights issues and reveal the processes by which powerful actors can come to see these grievances as legitimate claims. It is an important reminder of the dynamics of change and the ways in which rights frameworks and understandings are mutable.

In addition to drawing attention to the construction of rights – and of ‘making do’ – by marginalised individuals and groups, the papers highlight the role of institutions as brokers for and barriers to the realisation of rights. As discussed in several of the settings, institutional barriers and patriarchal mechanisms, that may also operate within plural judicial systems, coalesce to obstruct rather than facilitate the practical realisation of rights. In highlighting practical and epistemic entanglements that impinge on sexual and reproductive health rights, the papers shift the gaze from an exclusive focus on how groups and individuals negotiate rights in complex contexts, to the recognition that institutions themselves may inhibit the realisation of these rights. In this way, the papers draw attention to the intersections of international policy, national institutions and the messy realities in which people live and negotiate their lives and their rights.

The papers also contribute to a problematisation of concepts of rights drawn from international legal human rights framings. Particularly in national settings, where rights language has met with hostility, they raise two major questions. The first is an epistemic one. Where notions of self, personhood and entitlements diverge from the liberal individual model which to an extent underlies the international frameworks, what would a stronger focus on more collective and social concepts of rights and responsibilities, as against more individualised concepts of rights look like in the context of SRH? How can the potential pitfalls of cultural relativism that lurk behind this question be avoided? The danger, as Kabeer notes in her wide ranging review of gender and social justice approaches, is of endorsing behaviours that entrench gender and other forms of discrimination under the guise of the ‘collective’ [34]. The need is to explore further how and in which contexts concepts of more collectively defined rights and responsibilities can be reconciled with ones based in liberal jurisprudence. These papers suggest a developing research agenda of questions that would benefit from more systematic work from different contexts to illuminate this, for example:

• What are the key areas of personal and public concern that frame people’s thinking on SRH, sexuality, rights and responsibilities (for instance culture, religion, gender norms)?

• How do different cohorts (by generation, gender, and marital status), sexual minorities and stigmatised groups or individuals understand and negotiate concepts of rights and responsibilities, entitlements and wellbeing in relation to SRH?

• Who do people feel responsible for and why? What countermands ‘rights infringing behaviour’ and how do people negotiate solutions for themselves/others?

The second is a practical one. What are the implications for policy and application of rights-based approaches in SRH, given the often wide gap between the international and the local, and the endemic problems of the interpretation and implementation of international frameworks and conventions ‘on the ground’ in different contexts? How can we work to develop more nuanced, context-embedded responses? Here, the papers have useful suggestions to offer in terms of the need to pay close attention to diverse local gendered realities, culture and language, and ways of allying and working creatively with influential social and political actors.

Cutting across the epistemic and practical questions is a broader question about learning. Given that the ways in which different people understand and negotiate concepts of SRHR are embedded in gendered and cultural contexts, albeit fluid and evolving ones, what are the best ways to pool learning from diverse contexts and share promising practices on a comparative basis? It is likely that much of this learning takes place in informal ways and increasingly via new forms of social networking, such as the internet. This poses an interesting challenge to researchers and practitioners in developing forums that can influence the formal apparatus of rights framing.

## List of abbreviations

ICPD: International Conference on Population and Development; MPoA: Maputo Plan of Action; NGO: non-governmental organisation; RR RPC: Realising Rights Research Programme Consortium; SRH: Sexual and Reproductive Health; SRHR: Sexual and Reproductive Health and Rights; TAC: Treatment Action Campaign.

## Competing interests

The authors declare they have no competing interests.

## Authors’ contributions

HS wrote the first draft. EM, KH, ST and CU reviewed and contributed additional sections and revisions to the manuscript. All authors have seen and approved the final version.
